# 多项肺系统肿瘤标志物异常在晚期肺腺癌治疗中的作用

**DOI:** 10.3779/j.issn.1009-3419.2017.10.05

**Published:** 2017-10-20

**Authors:** 彦 彭, 燕 王, 学志 郝, 峻岭 李, 雨桃 刘, 宏羽 王

**Affiliations:** 100021 北京，中国医学科学院北京协和医学院肿瘤医院 Cancer Hospital Chinese Academy of Medical Sciences & Peking Union Medical College, Beijing 100021, China

**Keywords:** 肺腺癌, 肿瘤标志物, 远处转移, 维持治疗, 复发, Lung adenocarcinoma, Tumor marker, Distant metastasis, Maintenance therapy, Relapse

## Abstract

**背景与目的:**

肺癌的常用肿瘤标志物中，癌胚抗原（carcinoembryonic antigen, CEA）与糖类抗原125（carbohydrate antigen 125, CA125）、细胞角蛋白19片段（cytokeratin 19, CYFRA21-1）与鳞状细胞癌抗原（squamous carcinoma antigen, SCC）、神经元特异性烯醇化酶（neuron specific enolase, NSE）与胃泌素释放肽前体（pro-gastrin-releasing peptide, ProGRP）分别在肺腺癌、肺鳞状细胞癌和小细胞肺癌中有较高表达。本研究旨在通过对比多项肿瘤标志物异常（A组）和仅CEA和/或CA125异常（B组）的两组晚期肺腺癌患者，探讨多项肿瘤标志物异常在疗效评价和预测复发方面的价值。

**方法:**

纳入中国医学科学院肿瘤医院的Ⅳ期肺腺癌初治病例，回顾性分析其临床数据，包括临床特征、治疗前的血清肿瘤标志物水平、疗效及无进展生存期。

**结果:**

除CEA和CA125外，A组异常比率最高的肿瘤标志物是CYFRA21-1（93%），其次是NSE（36%）、SCC（13%）和ProGRP（12%）。多项肿瘤标志物异常的患者更易出现远处多部位转移（*P* < 0.001），治疗后的无进展生存期更短（中位时间5.3个月*vs* 7.3个月，*P*=0.016）。两组中进行维持治疗的患者均比未行维持治疗的患者复发风险低（*P*均 < 0.001）。

**结论:**

多项肿瘤标志物异常患者复发风险高，维持治疗可降低复发风险。

## Abstract (en)

**Background and objective:**

Among frequently-used tumor markers in lung cancer, carcinoembryonic antigen (CEA) and carbohydrate antigen 125 (CA125), cytokeratin 19 (CYFRA21-1) and squamous carcinoma antigen (SCC), neuron specific enolase (NSE) and pro-gastrin-releasing peptide (ProGRP) are respectively expressed highly in lung adenocarcinoma, lung squamous carcinoma and small cell lung cancer. By comparing patients with multiple increased tumor markers (group A) and patients with increase of CEA and/or CA125 (group B), this study aims to investigate the utility of multiple increased tumor markers in therapeutic evaluation and prediction of disease relapsing in patients with advanced lung adenocarcinoma.

**Methods:**

Patients with stage Ⅳ lung adenocarcinoma who receiving the first line chemotherapy in Cancer Hospital, Chinese Academy of Medical Sciences were enrolled and retrospectively analyzed. Clinical characteristic, serum tumor markers before chemotherapy, efficacy evaluation, progression-free survival (PFS) were analyzed.

**Results:**

Except CEA and CA125, the highest ratio of increased tumor markersin group A was CYFRA21-1 (93%), then was NSE (36%), SCC (13%) and ProGRP (12%). Patients with multiple increased tumor markers tend to have more distant metastasis (*P* < 0.001) and shorter PFS (median PFS 5.3 months *vs* 7.3 months, *P*=0.016). The relapse risk was lower in patients who accepted maintenance therapy than those who didn't accept maintenance therapy in both groups (*P* < 0.001).

**Conclusion:**

Patients with multiple increased tumor markers have high risk of relapse, and maintenance therapy can reduce relapse risk.

肺癌是目前我国乃至全世界发病率与死亡率最高的恶性肿瘤。根据生物学行为的不同，将肺癌分为非小细胞肺癌和小细胞肺癌。非小细胞肺癌占肺癌的80%，主要分为腺癌、鳞状细胞癌及大细胞癌等；小细胞肺癌约占肺癌的20%。目前常用于肺癌的肿瘤标志物有癌胚抗原（carcinoembryonic antigen, CEA），糖类抗原125（carbohydrate antigen 125, CA125），细胞角蛋白19片段（cytokeratin 19, CYFRA21-1），鳞状细胞癌抗原（squamous carcinoma antigen, SCC），神经元特异性烯醇化酶（neuron specific enolase, NSE）以及胃泌素释放肽前体（pro-gastrin-releasing peptide, ProGRP）。肺腺癌中常见到CEA及CA125的异常增高^[1, 2]^；CYFRA21-1和SCC在肺鳞状细胞癌中的阳性率最高^[3]^；NSE和ProGRP在小细胞肺癌中的表达最高^[4]^。在临床中经常可以发现一些肺腺癌患者除了CEA及CA125增高，还同时合并其他一项或多项肿瘤标志物升高。多项肿瘤标志物的升高在患者治疗中的提示作用方面资料相对较少。本研究分析中国医学科学院肿瘤医院治疗的晚期肺腺癌的数据，探讨多项肺系统肿瘤标志物异常在疗效评价和预测复发中的作用。

## 1 资料与方法

### 1.1 一般资料

收集自2014年6月-2016年6月在中国医学科学院肿瘤医院治疗的Ⅳ期肺腺癌初诊患者144例。所有患者经病理或细胞学，以及免疫组化确诊为肺腺癌。

### 1.2 治疗方案

所有患者均接受一线化疗，化疗方案的选择由临床医师按照美国国立综合癌症网络治疗指南并根据患者一般状况选择。

### 1.3 临床特征

通过查阅病历获得患者的临床基线特征及治疗数据。包括：患者性别、年龄、美国东部肿瘤协作组（Eastern Cooperative Oncology Group, ECOG）评分、吸烟情况、表皮生长因子受体（epidermal growth factor receptor, *EGFR*）基因及间变性淋巴瘤激酶（anaplastic lymphoma kinase, *ALK*）基因突变状态、肿瘤远处转移数量、一线化疗方案、疗效和一线治疗后疾病复发时间。

### 1.4 疗效评价及结果判定

按照实体瘤疗效评价标准（Response Evaluation Criteria in Solid Tumors, RECIST）1.1版^[5]^，分为完全缓解（complete response, CR）、部分缓解（partial response, PR）、疾病稳定（stable disease, SD）和疾病进展（progressive disease, PD）。无进展生存期（progression-free survival, PFS）为自首次化疗至首次PD或死亡时间。血清指标检测方法：CEA、CA125、CYFRA21-1及NSE为免疫电化学发光方法；SCC及ProGRP为免疫化学发光方法。正常值范围：CEA为0-5 ng/mL，CA125为0-35 U/mL，CYFRA21-1为0-3.3 ng/mL，SCC为0-1.5 ng/mL，NSE为0-18 ng/mL，ProGRP为0-50 pg/mL。所有患者均随访至一线治疗后疾病进展。

### 1.5 统计学方法

采用SPSS 22.0统计学软件进行数据的统计学处理。因标志物数据不符合正态分布，计量资料采用中位数及四分位数间距描述，计数资料采用例数及百分比描述。组间对比采用秩和检验、卡方检验和*Fisher’s*精确概率法，预后因素分析采用*Cox*回归分析。*P* < 0.05为差异有统计学意义。

## 2 结果

### 2.1 患者基线特征

共纳入144例患者，其中男性86例（59.7%），女性58例（40.3%）；年龄中位54.5岁（26岁-76岁）；化疗前ECOG评分均为0-2分；按照除CEA和/或CA125增高外是否合并其他肺系统肿瘤标志物增高将患者分为A、B两组，A组为疗前有多项肿瘤标志物增高患者，B组为疗前仅有CEA和/或CA125增高患者。本研究纳入A组患者100例，B组患者44例，两组患者在性别、年龄、疗前ECOG评分、吸烟情况、*EGFR*及*ALK*基因突变状态及是否进行维持治疗方面无差异（表 1）。除CEA及CA125外，A组异常肿瘤标志物中占比最多的是CYFRA21-1（93%），其次是NSE（36%）、SCC（13%）和ProGRP（12%）。67.0%的多项肿瘤标志物异常患者有多部位远处转移，这一比率在CEA和/或CA125增高的患者中仅为31.8%（*P* < 0.001）（表 2）。

**1:** 两组患者临床特征比较[*n* (%)] Comparison of clinical characteristic between two groups [*n* (%)]

Group	Multiple tumor markers increase (*n*=100)	CEA and/or CA125 increase (*n*=44)	Z/*χ*^2^	*P*	
Gender			0.706	0.401
Male	62 (62.0)	24 (54.5)		
Female	38 (38.0)	20 (45.5)		
Age (yr)			0.467	0.495
< 60	67 (67.0)	32 (72.7)		
≥60	33 (33.0)	12 (27.3)		
ECOG				0.260^*^
0	2 (2.0)	2 (4.5)		
1	93 (93.0)	42 (95.5)		
2	5 (5.0)	0 (0.0)		
Smoking history			1.598	0.206
No	57 (57.0)	30 (68.2)		
Current or former	43 (43.0)	14 (31.8)		
EGFR/ALK status			0.170	0.680
Wild/Unknown	60 (60.0)	28 (63.6)		
Mutant	40 (40.0)	16 (36.4)		
Maintenance therapy			-2.439	0.118
No	66 (66.0)	23 (52.3)		
Yes	34 (34.0)	21 (47.7)		
CEA: carcinoembryonic antigen; CA125: carbohydrate antigen 125; EGFR: epidermal growth factor receptor; ALK: anaplasticlymphoma kinase; ECOG: Eastern Cooperative Oncology Group. ^*^: *Fisher's* exact test.				

**2:** 两组患者肿瘤标志物水平、远处转移数目及疗效比较 Comparison of tumor markers (Md/Q) and number of distant metastasis and the best efficacy between two groups

Item	Multiple tumor markers increase (*n*=100)	CEA and/or CA125 increase (*n*=44)	Z/*χ*^2^	*P*	
Tumor markers (Md/Q)					
CEA (ng/mL)	22.62 (90.33)	13.95 (42.94)	-1.418	0.156	
CA125 (U/mL)	79.43 (155.61)	55.40 (114.84)	-1.932	0.053	
CYFRA21-1 (ng/mL)	9.18 (12.22)	2.33 (1.35)	-8.921	< 0.001	
SCC (ng/mL)	0.76 (0.84)	0.60 (0.57)	-1.352	0.177	
NSE (ng/mL)	17.54 (11.01)	12.22 (3.04)	-6.477	< 0.001	
ProGRP (pg/mL)	43.30 (19.83)	33.83 (15.18)	-3.240	0.001	
Number of distantmetastasis			15.368	< 0.001	
1	33 (33.0)	30 (68.2)			
≥2	67 (67.0)	14 (31.8)			
The best efficacy					
PR	44 (44.0)	22 (50.0)	0.443	0.506	
SD	41 (41.0)	17 (38.6)	0.071	0.790	
PD	15 (15.0)	5 (11.4)	0.338	0.561	
CYFRA21-1: cytokeratin 19; SCC: squamous carcinoma antigen; NSE: neuron specific enolase; ProGRP: pro-gastrin-releasing peptide; PR: partial response; SD: stable disease; PD: progressive disease.				

### 2.2 治疗方案

全组患者完成化疗的中位数为6周期（2周期-37周期），141例（97.9%）患者行含铂类（顺铂、卡铂及奈达铂）联合方案化疗，3例（2.1%）行单药化疗。化疗药物的选择上，培美曲塞占108例（75.0%），紫杉醇27例（18.8%），多西他赛4例（2.8%），吉西他滨3例（2.1%），长春瑞滨2例（1.3%）。23例（16.0%）患者在化疗的基础上联合了抗血管生成药物治疗（贝伐珠单抗及重组人血管内皮抑制素）。55例（38.2%）患者在联合化疗后继续维持治疗。

### 2.3 缓解率

一线化疗中最好疗效为PR者有66例（45.8%），SD有58例（40.3%），PD有20例（13.9%）。两组患者在各疗效组间无差异（表 2）。

### 2.4 复发情况

伴有多项肿瘤标志物异常的患者一线化疗中位PFS为5.3个月，仅CEA和/或CA125增高的患者中位PFS为7.3个月，将远处转移数目纳入*Cox*模型校正分析后，两组患者PFS体现出明显差异（*P*=0.016）（图 1）。单因素分析提示，两组中接受维持治疗的患者均较未行维持治疗的患者复发风险低（*P*均 < 0.001）（表 3）。

**1:**
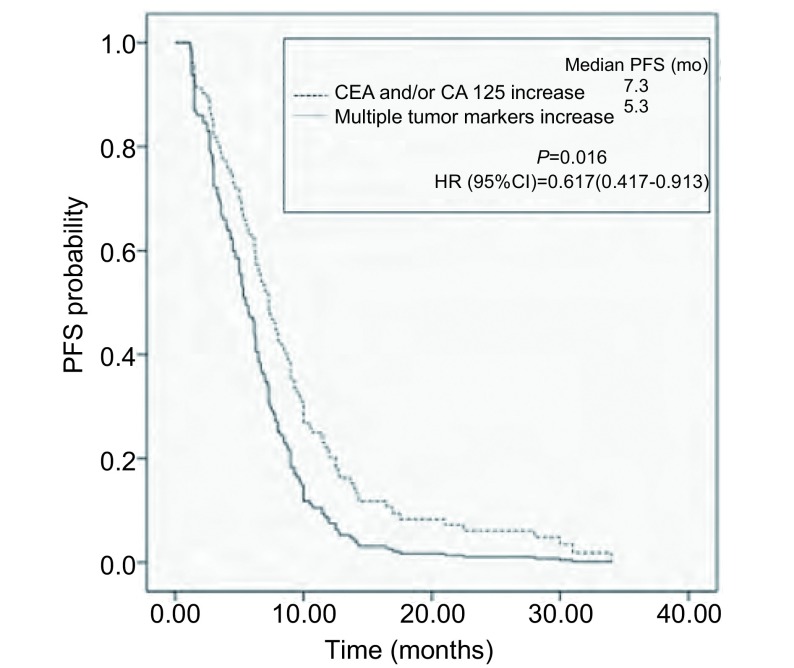
两组患者一线化疗PFS比较 Comparison of PFS of the first line chemotherapy between two groups. PFS: progression-free survival.

**3:** 两组患者复发风险的单因素分析 Prognostic factors for PFS of two groups by univariate analysis

Factors	Multiple tumor markers increase			CEA and/or CA125 increase	
	*P*	HR (95%CI)		*P*	HR (95%CI)
Gender (male *vs* female)	0.939	1.016 (0.672-1.538)		0.322	1.365 (0.737-2.526)
Age (yr)(≥60 *vs* < 60)	0.093	1.440 (0.941-2.204)		0.412	0.739 (0.359-1.522)
Smoking history (current or former *vs* no)	0.319	0.815 (0.544-1.219)		0.315	1.396 (0.728-2.677)
EGFR/ALK status (mutant *vs* wild/unknown)	0.061	1.475 (0.982-2.215)		0.917	0.967 (0.518-1.808)
Maintenance therapy (yes *vs* no)	< 0.001	0.446 (0.287-0.694)		< 0.001	0.279 (0.144-0.540)
Number of distant metastasis (≥2 *vs* 1)	0.592	1.122 (0.736-1.711)		0.528	1.231 (0.646-2.344)

## 3 讨论

本研究中多项肿瘤标志物异常组除CEA及CA125外最常见的异常标志物为CYFRA21-1和NSE。CYFRA21-1存在于上皮来源的肿瘤细胞的胞浆中，在非小细胞肺癌中的表达高于小细胞肺癌，阳性率以鳞癌最高；在非小细胞肺癌，尤其是肺鳞癌中的诊断和预后中的作用得到证实。NSE是神经内分泌细胞特有的烯醇化酶的同工酶，在小细胞肺癌中有过量表达。本研究中A组有高达93%的患者出现CYFRA21-1的异常增高，提示对于CYFRA21-1在肺腺癌中的应用值得更多关注。Park等^[6]^对可手术切除的肺腺癌患者进行观察，发现高CYFRA21-1水平的患者分期更晚，肺部肿瘤更大，分化更低，5年生存率更低，可作为可手术切除的肺腺癌的预后因素。Cabreraalarcon等^[7]^发现在非小细胞肺癌患者中，Ⅳ期患者的CYFRA21-1和NSE水平与其他期别差异显著。Zhang等^[8]^发现CYFRA21-1和NSE可作为肺癌的独立预后因素，CYFRA21-1在肺腺癌中与肿瘤的远处转移密切相关。本研究中A组患者远处多部位转移率明显高于B组患者，提示多项肿瘤标志物异常更易出现广泛的远处转移，与既往研究相符。

Pang等^[9]^研究发现CYFRA21-1较高的非小细胞肺癌患者的化疗疗效较差，本研究中两组患者在化疗的疗效方面无明显差异，仍需积累更多样本进一步观察。多项肿瘤标志物异常的患者一线化疗后PFS时间短于仅CEA和/或CA125异常的患者，考虑到两组患者在肿瘤远处转移的多寡上有差异，进行校正分析后，PFS仍有统计学差异，提示多项肿瘤标志物异常的肺腺癌患者近期复发风险更高。既往研究亦报道过较高CEA、CA125和CYFRA21-1水平的非小细胞肺癌患者化疗后PFS较短^[2]^。CYFRA21-1在肺腺癌的疗效评价及近期复发方面的作用值得进一步探讨。

既往研究证实IIIb期和Ⅳ期非小细胞肺癌患者在一线化疗后进行培美曲塞维持治疗可明显延长PFS^[10]^。本研究两组患者多接受4个-6个周期的含铂类联合方案一线化疗，部分患者进行了后续单药维持治疗，进行维持治疗的大部分患者接受了培美曲塞治疗。单因素分析提示两组接受维持治疗的患者复发风险均小于未接受维持治疗的患者，同既往文献相符。

本项研究的不足之处在于为回顾性，样本量仍需进一步积累，以便得到更稳健的结果。多项肿瘤标志物异常的晚期肺腺癌患者更易出现多发远处转移，复发风险高，维持治疗可降低复发风险，提示在临床工作中可考虑对这部分患者适当进行更积极地维持治疗。
